# Supplementary material for: Gene expression profiling unveils the temporal dynamics of CIGB-300-regulated transcriptome in AML cell lines
Source: BMC Genomics. 2023 Jul 4;24:373. doi: 10.1186/s12864-023-09472-5 (PMC10318747; doi:10.1186/s12864-023-09472-5)
Supplement: Supplementary file 1 — Additional file 1: Figure S1. Diagnostic plots in control cells or CIGB-300 treated OCI-AML3 (O) and HL-60 (H) samples for 3h and 30min. (A) One dimensional hierarchical clustering of all samples (replicates 1, 2, 3), (B) ANOVA R-squared (^2) of covariates (Cell, Time and Treatment) in % (C), Multidimensional Scaling (MDS) of filtered data. Samples from HL60 (H) and OCI-AML3 (O) Untreated or treated with the peptide CIGB-300 for 30min and 3h are indicated. Figure S2. Top 100 differentially up-regulated genes by CIGB-300. (A) Unsupervised Heatmap from the Top 100 most differentially up-expressed genes in HL-60 and OCI-AML3. (B) Pathways analysis in Top 100 up-regulated genes using Enrichr. Figure S3. Top 100 enriched ontology clusters across AML cell lines treated with CIGB-300 for 30min and 3h using Metascape. The most 100 enriched biological processes (-log10(P)) in HL-60 (H) or OCI-AML3 (OCI) after CIGB-300 treatment for 30min and 3h are shown. Figure S4. MCODE in network using Metascape. MCODE was used to identify neighborhoods where proteins are densely connected in network. Network nodes are displayed as pies, where colors in sectors represent the treatment groups according to the legend at the draw corner for HL-60 (H) and OCI-AML3 (OCI) treated with CIGB-300 for 30min and 3h. Figure S5. Predicted Transcription factors from DEGs. TF predicted from regulatory motifs in DEGs in HL-60 and OCI-AML3 after 30min and 3h of CIGB-300 treatment are shown in both panels of the Figure. The Cytoscape on-net analysis plugin iRegulon was used and a NES>3.0 was selected. Figure S6. qPCR validation of genes related to differentiation modulated by CIGB-300. HL-60 and OCI-AML3 cells were treated with 40 μM of CIGB-300 for 30min and 3h. Histogram bars indicate relative mRNA levels ± standard error with respect to a time-matched untreated control, for two independent experiments analyzed in triplicate. All genes were normalized with ABL1, DDX5, and GAPDH genes. Asterisks represent [file 12864_2023_9472_MOESM1_ESM.pdf]

Gene Expression Profiling Unveils the Temporal Dynamics of CIGB-300-Regulated Transcriptome in AML Cell lines

Dania Vázquez-Blomquist [1,*], Ailyn C. Ramón [2], Mauro Rosales [2,3], George V. Pérez [2], Ailenis Rosales [3], Daniel Palenzuela [1], Yasser Perera [2,4,*] and Silvio E. Perea [2,*]

Supplementary Figures and Tables Captions

Figure S1. Diagnostic plots in control cells or CIGB-300 treated OCI-AML3 (O) and HL-60 (H) samples for 3h and 30min. (A) One dimensional hierarchical clustering of all samples (replicates 1, 2, 3), (B) ANOVA R-squared (^2) of covariates (Cell, Time and Treatment) in % (C), Multidimensional Scaling (MDS) of filtered data. Samples from HL60 (H) and OCI-AML3 (O) Untreated or treated with the peptide CIGB-300 for 30min and 3h are indicated.

Figure S2. Top 100 differentially up-regulated genes by CIGB-300. (A) Unsupervised Heatmap from the Top 100 most differentially up-expressed genes in HL-60 and OCI-AML3. (B) Pathways analysis in Top 100 up-regulated genes using Enrichr.

Figure S3. Top 100 enriched ontology clusters across AML cell lines treated with CIGB-300 for 30min and 3h using Metascape. The most 100 enriched biological processes (-log10(P)) in HL-60 (H) or OCI-AML3 (OCI) after CIGB-300 treatment for 30min and 3h are shown.

Figure S4. MCODE in network using Metascape. MCODE was used to identify neighborhoods where proteins are densely connected in network. Network nodes are displayed as pies, where colors in sectors represent the treatment groups according to the legend at the draw corner for HL-60 (H) and OCI-AML3 (OCI) treated with CIGB-300 for 30min and 3h.

Figure S5. Predicted Transcription factors from DEGs. TF predicted from regulatory motifs in DEGs in HL-60 and OCI-AML3 after 30min and 3h of CIGB-300 treatment are shown in both panels of the Figure. The Cytoscape on-net analysis plugin iRegulon was used and a NES>3.0 was selected.

Figure S6. qPCR validation of genes related to differentiation modulated by CIGB-300. HL-60 and OCI-AML3 cells were treated with 40 µM of CIGB-300 for 30min and 3h. Histogram bars indicate relative mRNA levels ± standard error with respect to a time-matched untreated control, for two independent experiments analyzed in triplicate. All genes were normalized with ABL1, DDX5, and GAPDH genes. Asterisks represent statistically significant changes ($p < 0.05$) by REST 2009.

Figure S7. qPCR validation of the cell cycle related gene CDKN1A/P21 gene modulated by CIGB-300. HL-60 and OCI-AML3 cells were treated with 40 µM of CIGB-300 for 30min, 2h and 8h. Histogram bars indicate relative mRNA levels ± standard error with respect to a time-matched untreated control, for two independent experiments analyzed in triplicate. All genes were normalized with ABL1, DDX5, and GAPDH genes. Asterisks represent statistically significant changes ($p < 0.05$) by REST 2009.

Table S1 (Additional File 1). Oligonucleotides used for qPCR amplifications. Information of Gene name, oligonucleotide identifiers (ID), Sequence 5´ to 3´ (Sequence 5'…3'), number of bases (# Bases) and Function (Reference or Biomarker) is provided. All were synthesized in Oligonucleotide Synthesis Group (CIGB, Havana).

Table S2 (Additional File 2). DEGs for the treatment groups comparisons (p< 0.01;|FC|>=1.5). In independent sheets we have HL-60 *vs* Untreated control 30min & 3h, and OCI-AML3 *vs* Untreated control 30min & 3h. Column order: # number of gene, Gene SYMBOL, Fold Change, P value, adjusted (adj) P value, ENTREZ ID, Gene Name.

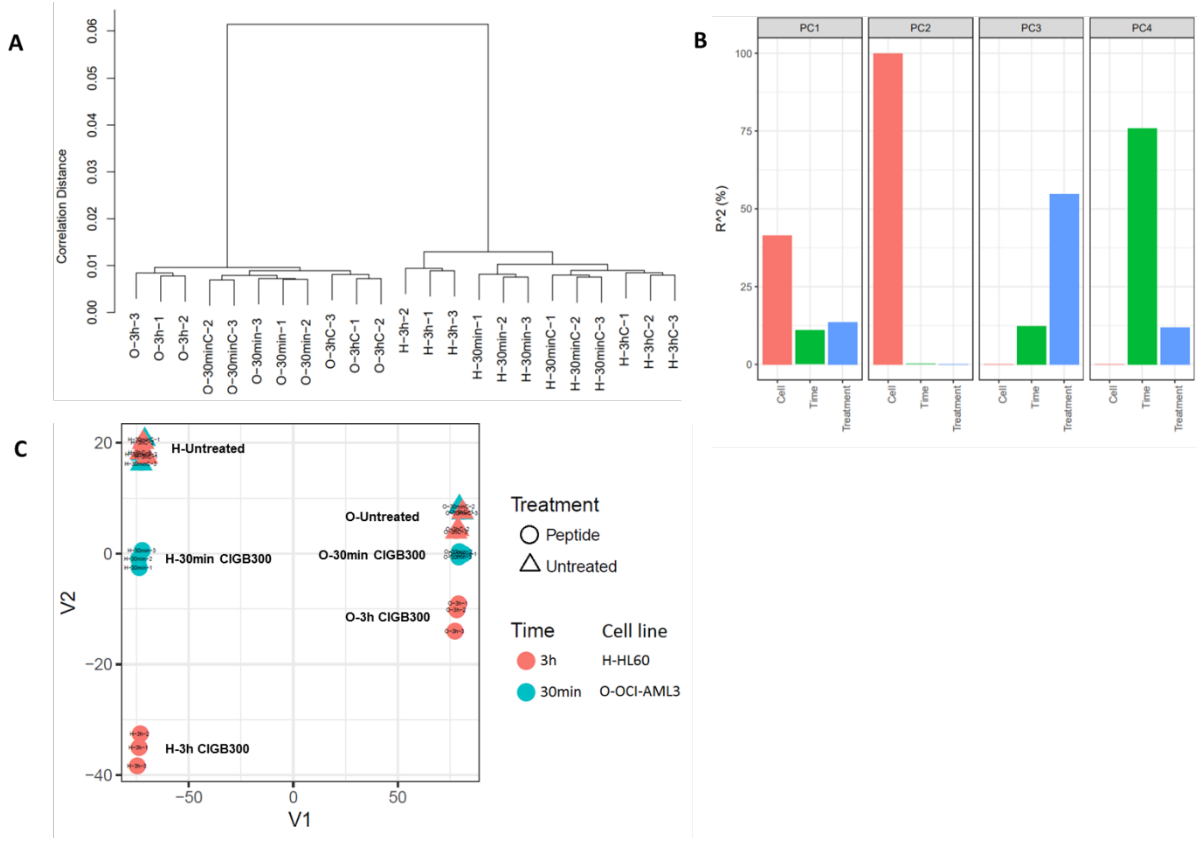
A
Correlation Distance
0.00
0.01
0.02
0.03
0.04
0.05
0.06
O−3h−3
O−3h−1
O−3h−2
O−30minC−2
O−30minC−3
O−30min−3
O−30min−1
O−30min−2
O−3hC−3
O−3hC−1
O−3hC−2
H−3h−2
H−3h−1
H−3h−3
H−30min−1
H−30min−2
H−30min−3
H−30minC−1
H−30minC−2
H−30minC−3
H−3hC−1
H−3hC−2
H−3hC−3
B
PC1
PC2
PC3
PC4
R^2 (%)
100
75
50
25
0
Cell
Time
Treatment
C
H-Untreated
O-Untreated
H-30min CIGB300
O-30min CIGB300
O-3h CIGB300
H-3h CIGB300
V2
V1
20
0
−20
−40
−50
0
50
Treatment
Peptide
Untreated
Time
3h
30min
Cell line
H-HL60
O-OCI-AML3

Figure S1.

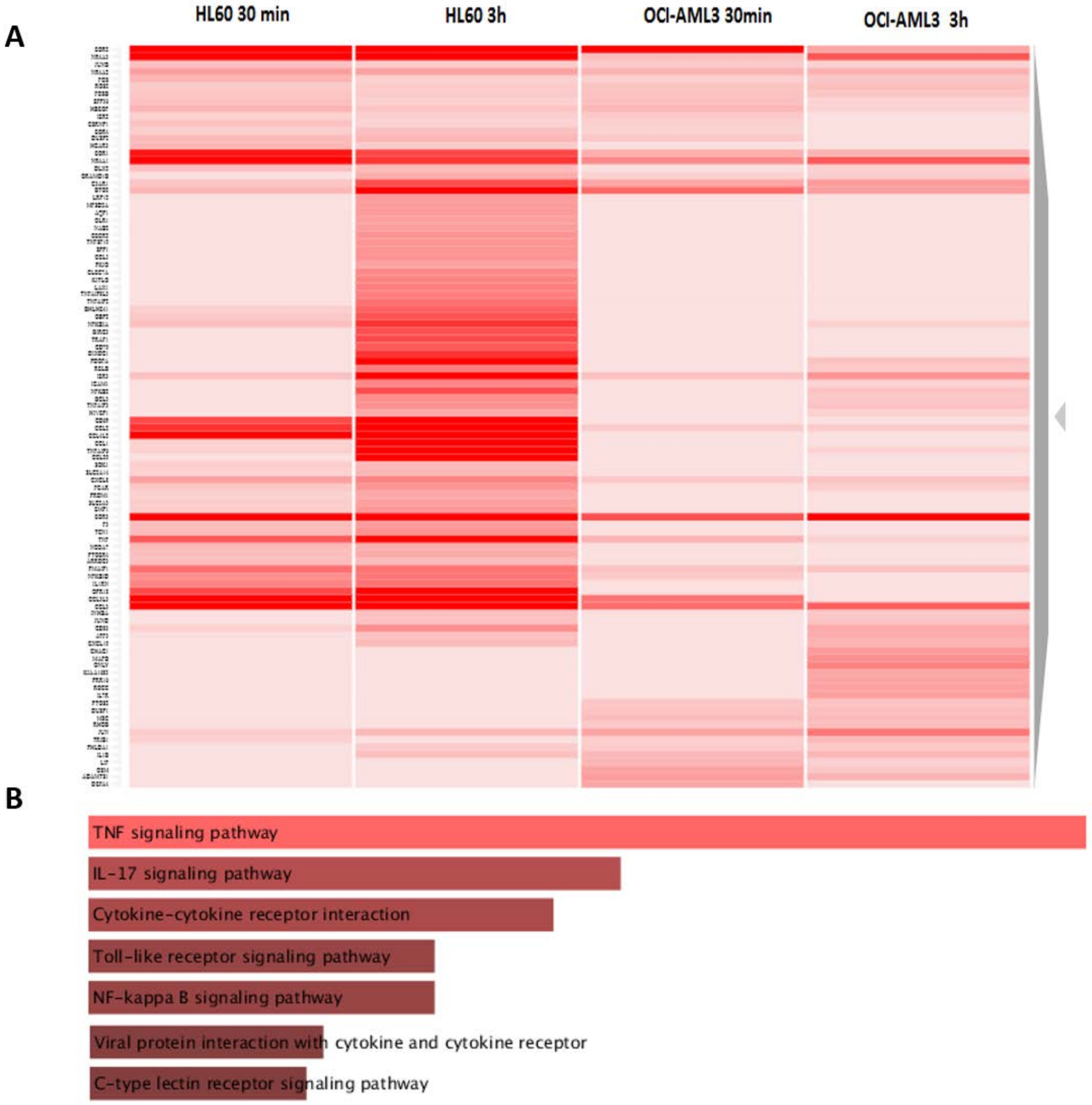
A
HL60 30 min
HL60 3h
OCI-AML3 30min
OCI-AML3 3h
B
TNF signaling pathway
IL-17 signaling pathway
Cytokine-cytokine receptor interaction
Toll-like receptor signaling pathway
NF-kappa B signaling pathway
Viral protein interaction with cytokine and cytokine receptor
C-type lectin receptor signaling pathway

Figure S2.

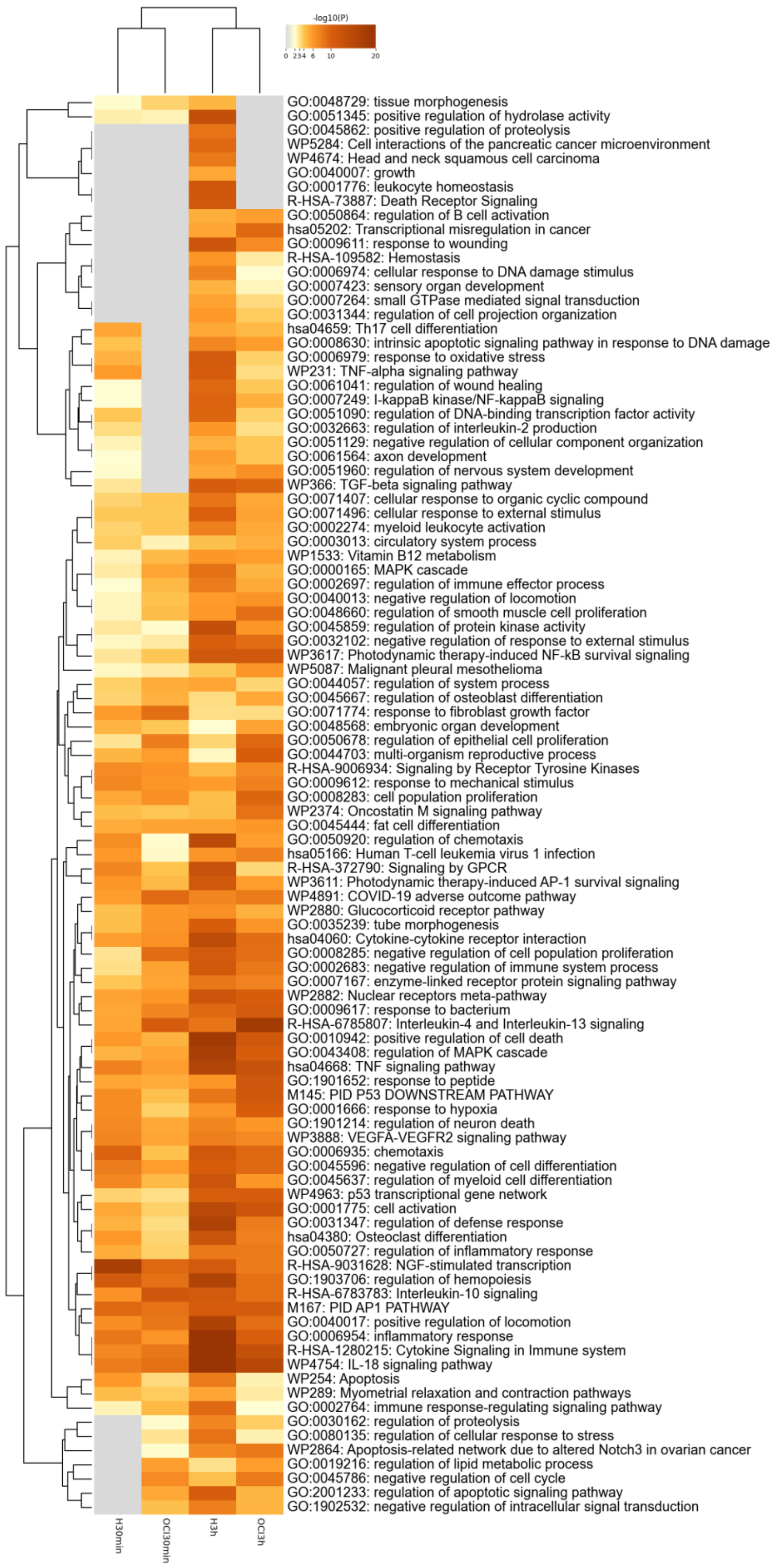
-log10(P)
0 2 3 4 6 10 20
GO:0048729: tissue morphogenesis
GO:0051345: positive regulation of hydrolase activity
GO:0045862: positive regulation of proteolysis
WP5284: Cell interactions of the pancreatic cancer microenvironment
WP4674: Head and neck squamous cell carcinoma
GO:0040007: growth
GO:0001776: leukocyte homeostasis
R-HSA-73887: Death Receptor Signaling
GO:0050864: regulation of B cell activation
hsa05202: Transcriptional misregulation in cancer
GO:0009611: response to wounding
R-HSA-109582: Hemostasis
GO:0006974: cellular response to DNA damage stimulus
GO:0007423: sensory organ development
GO:0007264: small GTPase mediated signal transduction
GO:0031344: regulation of cell projection organization
hsa04659: Th17 cell differentiation
GO:0008630: intrinsic apoptotic signaling pathway in response to DNA damage
GO:0006979: response to oxidative stress
WP231: TNF-alpha signaling pathway
GO:0061041: regulation of wound healing
GO:0007249: I-kappaB kinase/NF-kappaB signaling
GO:0051090: regulation of DNA-binding transcription factor activity
GO:0032663: regulation of interleukin-2 production
GO:0051129: negative regulation of cellular component organization
GO:0061564: axon development
GO:0051960: regulation of nervous system development
WP366: TGF-beta signaling pathway
GO:0071407: cellular response to organic cyclic compound
GO:0071496: cellular response to external stimulus
GO:0002274: myeloid leukocyte activation
GO:0003013: circulatory system process
WP1533: Vitamin B12 metabolism
GO:0000165: MAPK cascade
GO:0002697: regulation of immune effector process
GO:0040013: negative regulation of locomotion
GO:0048660: regulation of smooth muscle cell proliferation
GO:0045859: regulation of protein kinase activity
GO:0032102: negative regulation of response to external stimulus
WP3617: Photodynamic therapy-induced NF-kB survival signaling
WP5087: Malignant pleural mesothelioma
GO:0044057: regulation of system process
GO:0045667: regulation of osteoblast differentiation
GO:0071774: response to fibroblast growth factor
GO:0048568: embryonic organ development
GO:0050678: regulation of epithelial cell proliferation
GO:0044703: multi-organism reproductive process
R-HSA-9006934: Signaling by Receptor Tyrosine Kinases
GO:0009612: response to mechanical stimulus
GO:0008283: cell population proliferation
WP2374: Oncostatin M signaling pathway
GO:0045444: fat cell differentiation
GO:0050920: regulation of chemotaxis
hsa05166: Human T-cell leukemia virus 1 infection
R-HSA-372790: Signaling by GPCR
WP3611: Photodynamic therapy-induced AP-1 survival signaling
WP4891: COVID-19 adverse outcome pathway
WP2880: Glucocorticoid receptor pathway
GO:0035239: tube morphogenesis
hsa04060: Cytokine-cytokine receptor interaction
GO:0008285: negative regulation of cell population proliferation
GO:0002683: negative regulation of immune system process
GO:0007167: enzyme-linked receptor protein signaling pathway
WP2882: Nuclear receptors meta-pathway
GO:0009617: response to bacterium
R-HSA-6785807: Interleukin-4 and Interleukin-13 signaling
GO:0010942: positive regulation of cell death
GO:0043408: regulation of MAPK cascade
hsa04668: TNF signaling pathway
GO:1901652: response to peptide
M145: PID P53 DOWNSTREAM PATHWAY
GO:0001666: response to hypoxia
GO:1901214: regulation of neuron death
WP3888: VEGFA-VEGFR2 signaling pathway
GO:0006935: chemotaxis
GO:0045596: negative regulation of cell differentiation
GO:0045637: regulation of myeloid cell differentiation
WP4963: p53 transcriptional gene network
GO:0001775: cell activation
GO:0031347: regulation of defense response
hsa04380: Osteoclast differentiation
GO:0050727: regulation of inflammatory response
R-HSA-9031628: NGF-stimulated transcription
GO:1903706: regulation of hemopoiesis
R-HSA-6783783: Interleukin-10 signaling
M167: PID AP1 PATHWAY
GO:0040017: positive regulation of locomotion
GO:0006954: inflammatory response
R-HSA-1280215: Cytokine Signaling in Immune system
WP4754: IL-18 signaling pathway
WP254: Apoptosis
WP289: Myometrial relaxation and contraction pathways
GO:0002764: immune response-regulating signaling pathway
GO:0030162: regulation of proteolysis
GO:0080135: regulation of cellular response to stress
WP2864: Apoptosis-related network due to altered Notch3 in ovarian cancer
GO:0019216: regulation of lipid metabolic process
GO:0045786: negative regulation of cell cycle
GO:2001233: regulation of apoptotic signaling pathway
GO:1902532: negative regulation of intracellular signal transduction
H30min
OCI30min
H3h
OCI3h

Figure S3.

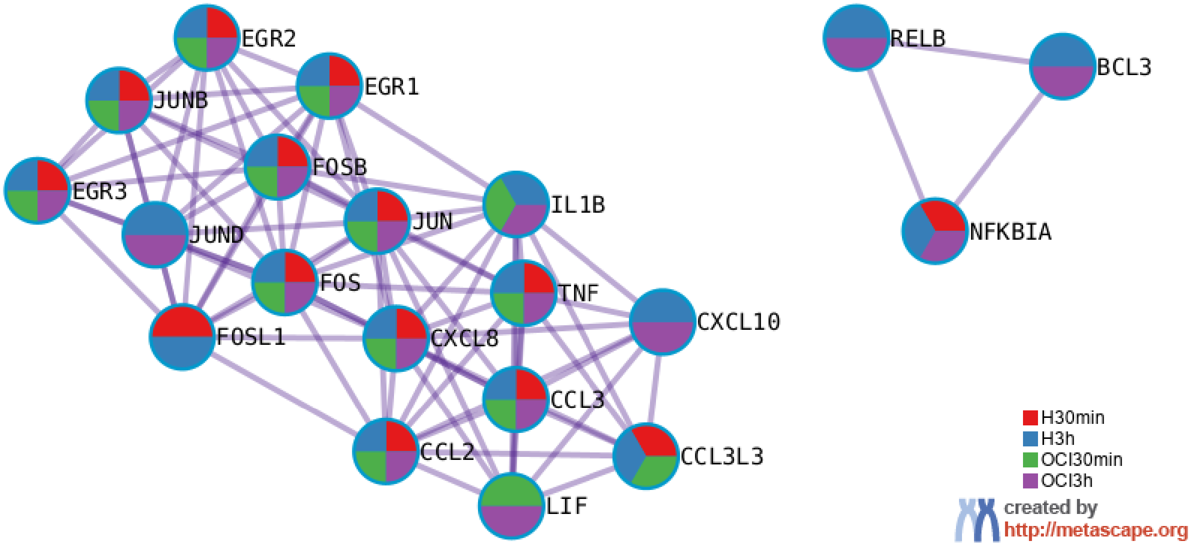
EGR2
EGR1
JUNB
FOSB
EGR3
JUND
JUN
IL1B
FOS
TNF
FOSL1
CXCL8
CXCL10
CCL3
CCL2
CCL3L3
LIF
RELB
BCL3
NFKBIA
H30min
H3h
OCI30min
OCI3h
created by
http://metascape.org

Figure S4.

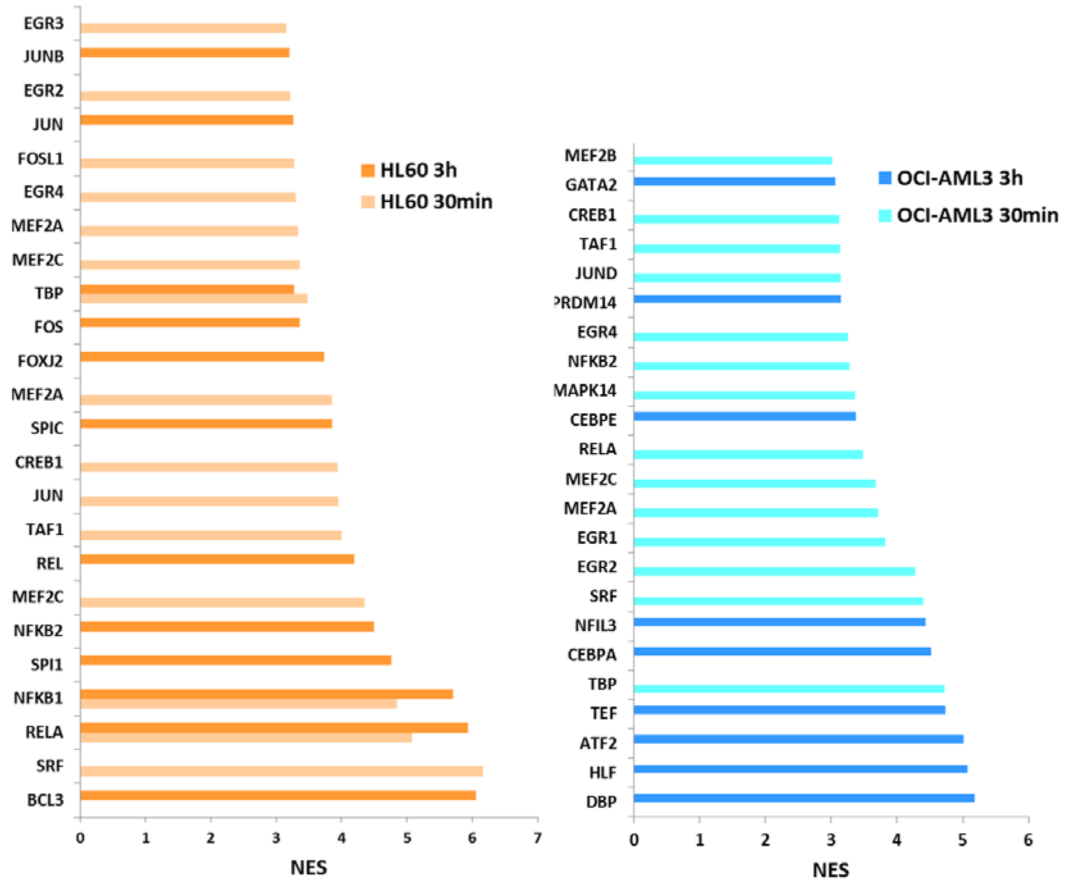
HL60 3h
HL60 30min
EGR3
JUNB
EGR2
JUN
FOSL1
EGR4
MEF2A
MEF2C
TBP
FOS
FOXJ2
MEF2A
SPIC
CREB1
JUN
TAF1
REL
MEF2C
NFKB2
SPI1
NFKB1
RELA
SRF
BCL3
0
1
2
3
4
5
6
7
NES
OCI-AML3 3h
OCI-AML3 30min
MEF2B
GATA2
CREB1
TAF1
JUND
PRDM14
EGR4
NFKB2
MAPK14
CEBPE
RELA
MEF2C
MEF2A
EGR1
EGR2
SRF
NFIL3
CEBPA
TBP
TEF
ATF2
HLF
DBP
0
1
2
3
4
5
6
NES

Figure S5.

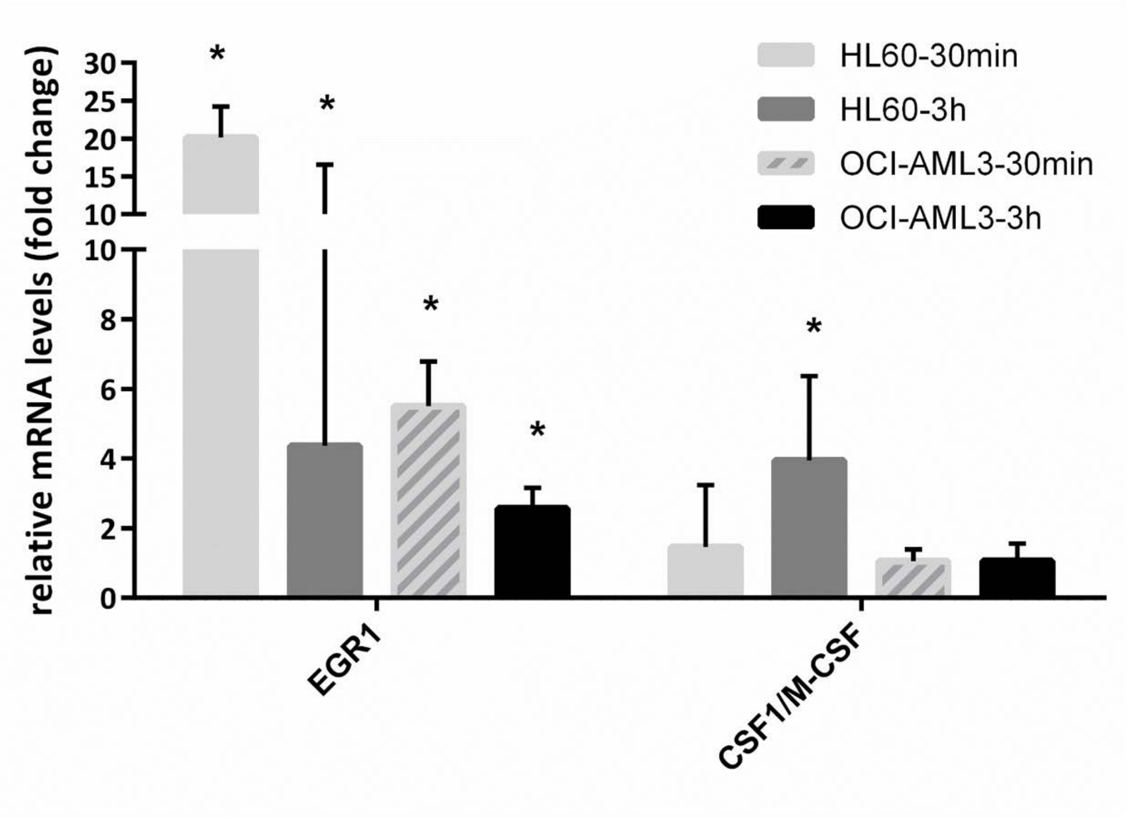

relative mRNA levels (fold change)
30
25
20
15
10
10
8
6
4
2
0
*
*
*
*
*
HL60-30min
HL60-3h
OCI-AML3-30min
OCI-AML3-3h
EGR1
CSF1/M-CSF

Figure S6.

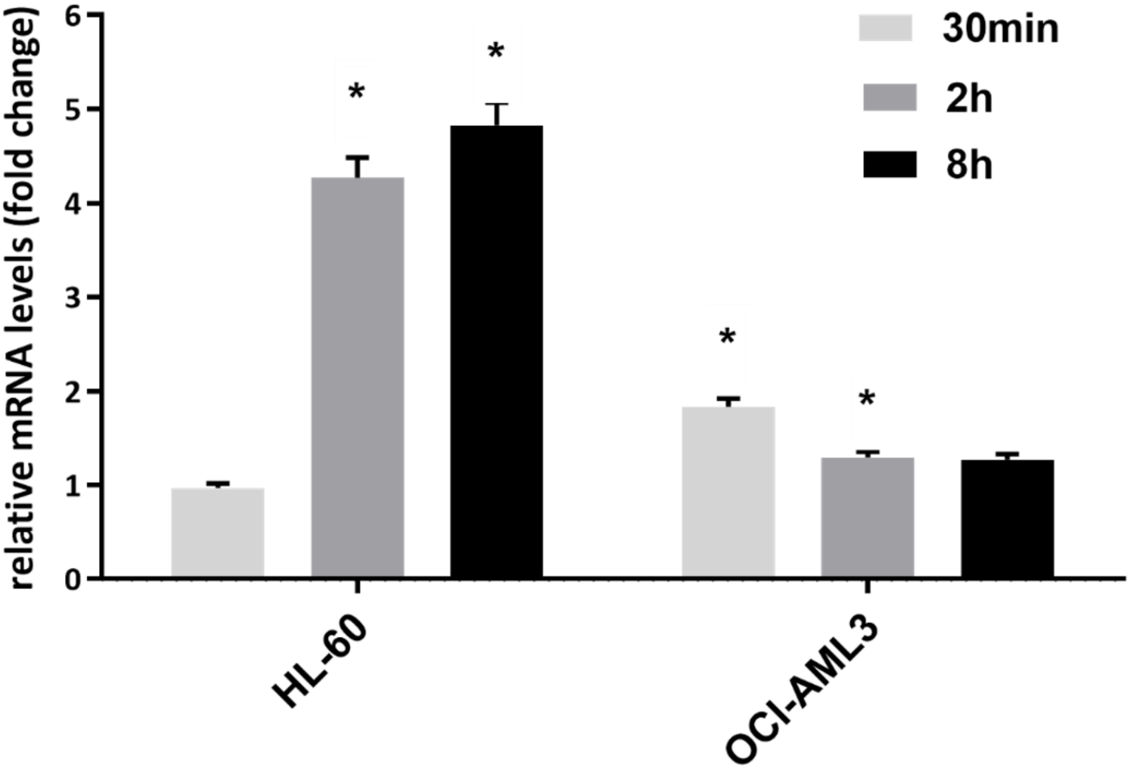
relative mRNA levels (fold change)
6
5
4
3
2
1
0
30min
2h
8h
*
*
*
*
HL-60
OCI-AML3

Figure S7.

Table S1 (Additional File 1).

Gene Name	ID	Sequence 5'…3'	# Bases	Function
ABL Proto-Oncogene 1, Non-Receptor Tyrosine Kinase	ABL1-F	ACGTCTGGGCATTTGGAGTATT	22	**Reference**
	ABL1-R	CTCCATGCGGTAGTCCTTCTCT	22	
DEAD (Asp-Glu-Ala-Asp) Box Helicase 5	DDX5-F	TAGAGGTCACAACTGCCCGAAG	22	**Reference**
	DDX5-R	GGCCATCCCTGAGCTTGAATAG	22	
Glyceraldehyde-3-Phosphate Dehydrogenase	GAPDH-F	GTCCACCACCCTGTTGCTGTAG	22	**Reference**
	GAPDH-R	ACTTCAACAGCGACACCCACTC	22	
Cyclin Dependent Kinase Inhibitor 1A (P21)	CDKN1A-F	CTTCAGTACCCTCTCAGCTCCA	22	**Biomarker**
	CDKN1A-R	AACTAGGGTGCCCTTCTTCTTG	22	
Early growth response 1	EGR1-F	CTTCAACCCTCAGGCGGACA	20	**Biomarker**
	EGR1-R	AAAGACTCTGCGGTCAGGTG	20	
Growth arrest and DNA damage inducible beta	GADD45B-F	ATCAACATCGTGCGGGTGTC	20	**Biomarker**
	GADD45B-R	GTGTGAGGGTTCGTGACCAG	20	
Mitogen-activated protein kinase kinase kinase 8	MAP3K8-F	GGCGTGTAAACTGATCCCAGTA	22	**Biomarker**
	MAP3K8-R	CCACAGGACTGCGCCATACA	20	
Colony stimulating factor 1 (CSF1)/M-CSF	MCSF-F	GTTGGTCTGTCTCCTGGCGA	20	**Biomarker**
	MCSF-R	TGGCACGAGGTCTCCATCTG	20	
Nuclear factor kappa B subunit 1 /p50	NFKB1-F	AGCCCAGTGAAGACCACCTCTC	22	**Biomarker**
	NFKB1-R	AAAGCTGAGTTTGCGGAAGGAT	22	
Nuclear factor kappa B subunit 2 /p52	NFKB2-F	AACCTCACCAACCACCTGCA	20	**Biomarker**
	NFKB2-R	AGTCTCCATGCCGATCCAGC	20	
NFKB inhibitor alpha /IKBA	NFKBIA-F	TGACTCAGTCCTGCACCACC	20	**Biomarker**
	NFKBIA-R	CCAAAAGCTCCACGATGCCC	20	
RELA proto-oncogene	RelA-F	CTGAATGCTGTGCGGCTCTG	20	**Biomarker**
	RelA-R	TCGGCAGATCTTGAGCTCGG	20	
REL proto-oncogene/c-REL	RelC-F	ACCTGGTCTCCTCGGTTCAA	20	**Biomarker**
	RelC-R	ACTTGAGATGGGCCCAGGTG	20	
TNF alpha	TNFA- F	AAGGACACCATGAGCACTGAAA	22	**Biomarker**
	TNFA- R	GAGAAGAGGCTGAGGAACAAGC	22	
TNF alpha induced protein 3 (A20)	TNFAIP3-F	GCGCTGAAAACGAACGGTGA	20	**Biomarker**
	TNFAIP3-R	ACAGCGCCTTCCTCAGTACC	20	
